# Anesthesiology Articles Published in 2020: A Review and Characterization of COVID-19 Versus Non-COVID-19 Publications in Top Anesthesiology Journals

**DOI:** 10.7759/cureus.23943

**Published:** 2022-04-08

**Authors:** Jamal K Egbaria, Alexander M Kofskey, Carter J Boyd, Brant Wagener

**Affiliations:** 1 Anesthesiology and Perioperative Medicine, University of Alabama at Birmingham, Birmingham, USA; 2 Medical School, University of Alabama at Birmingham Heersink School of Medicine, Birmingham, USA; 3 Hansjörg Wyss Department of Plastic Surgery, New York University (NYU) Langone, New York, USA

**Keywords:** coronavirus disease 2019 (covid-19), acute respiratory distress syndrome (ards), altmetric attention score (aas), severe acute respiratory syndrome coronavirus 2 (sars-cov-2), anesthesia literature

## Abstract

The coronavirus disease 2019 (COVID-19) pandemic has had a significant impact on the practice of medicine worldwide, particularly in anesthesiology. As the clinical realm has rapidly adjusted to the realities of the pandemic, anesthesiology literature has also changed significantly to reflect this. The purpose of this study was to characterize the effects the COVID-19 pandemic has had on anesthesiology literature. Specifically, it was hypothesized that the COVID-19-related literature in the anesthesiology community would gain more interest than non-COVID-19-related articles. A total of 15 anesthesiology-related journals with the highest impact factor in 2019, according to the Journal Citation Reports (JCR),* *were selected for data collection. An advanced PubMed search identified 5,722 COVID-19-related articles published by these journals in 2020. Next, articles with titles including “corona,” “COVID,” “COVID-19,” “pandemic,” “SARS,” or “SARS-CoV-2” were selected for inclusion in the study, which resulted in 676 (12%) articles. A Kruskal-Wallis test was used to assess the Altmetric score, which is a weighted calculation of the attention an article receives online, for COVID-19 versus non-COVID-19 articles. Articles were then further characterized across multiple different variables, including country of origin, month published, type of article, and subspecialty of anesthesiology it pertained to. Of the 15 journals investigated, 676 (12%) articles of the 5,722 total articles published were found to be COVID-19-related material. The majority of the articles were found to be published in April (18%), May (19.5%), and June (14%). The majority of these articles were related either to general anesthesia (operating room anesthesiology that is not tied to a particular subspecialty fellowship track) (48%) or critical care (39%). By article type, most were determined to be editorial (71%) in nature, followed by original research articles (21%), of which most were cross-sectional (55%) studies. When compared with non-COVID-19-related articles, COVID-19-related articles had a significantly greater Altmetric score (29.518 versus 8.6333, p < 0.001). Of the COVID-19-related articles, original articles had the greatest Altmetric score, when compared to editorials and guidelines (54.794 versus 20.777 versus 40.643, p < 0.002). The response of the academic anesthesiology community to the COVID-19 pandemic was strong and timely, with a particularly strong focus on critical care anesthesia. The impact of the pandemic was strongly felt by the anesthesiology community, and their timely response served to guide our country and world through an incredibly challenging time. The pandemic highlighted the value of anesthesiologists worldwide, not only in the operating room setting but particularly as critical care physicians.

## Introduction and background

The first known described outbreak of severe acute respiratory syndrome coronavirus 2 (SARS-CoV-2) and coronavirus disease 2019 (COVID-19) in the literature occurred in Wuhan, China, in December 2019 [1]. The spread of the virus to the United States was first documented on January 20, 2020, and by the end of the month, nearly 10,000 cases had been reported worldwide [2]. As the COVID-19 pandemic progressed, approximately 3.4 million deaths were reported as of April 2021 [3]. The COVID-19 pandemic placed a unique challenge on the medical community, with the additional responsibility for the academic medical community to produce data and high-impact research to guide clinicians through the pandemic.

Anesthesiologists have assumed a unique role during the COVID-19 pandemic [4]. As the practice of anesthesiology often requires airway manipulation and management of critically ill patients, anesthesia providers are at a higher risk of interaction and exposure to the COVID-19 virus [5,6]. Patients infected with SARS-CoV-2 often face complications such as acute respiratory distress syndrome (ARDS), septic shock, and multisystem organ failure, all of which can lead to rapid death and are managed actively by anesthesiologists in healthy and critically ill patients [7,8]. As such, the pandemic response has relied on the academic anesthesiology community and their ability to publish timely studies that would direct the management of patients with COVID-19.

As the clinical realm shifted to respond to this pandemic, one would expect that anesthesiology literature changed significantly to reflect this. The purpose of this study was to characterize the effects the COVID-19 pandemic had on anesthesiology literature. Specifically, it was hypothesized that COVID-19-related literature in the anesthesiology community would gain more interest than non-COVID-19-related articles, as measured by the Altmetric Attention Score (AAS), which is a surrogate of article interest, influence, and distribution both online and through other mediums [9-12]. Additionally, we hypothesized that articles related to critical care within the anesthesiology literature would garner more interest than other articles.

This article was previously presented as a poster at the 2021 ASA Anesthesiology annual meeting on October 9, 2021.

## Review

Materials and methods

Fifteen anesthesiology-related journals with the highest impact factor in 2019, according to the Journal Citation Reports (JCR), were selected for data collection. An advanced PubMed search identified 5,722 articles published by these journals in 2020. Subsequently, articles were screened for content to divide these articles into two cohorts. Articles including mention of “COVID,” “SARS,” “pandemic,” “corona,” “COVID-19,” “2019 nCoV,” “2019 novel coronavirus,” or “SARS-CoV-2” were selected for inclusion in the COVID-19-related cohort, which resulted in 676 (12%) articles. The remaining articles comprised the non-COVID-19 article cohort. For all articles, the AAS was determined on April 1, 2021, using the Altmetric bookmarklet tool. The AAS is a tool that measures the dissemination of a research publication through social media, news outlets, and other media. The AAS is a cumulative score derived from contributions from numerous online media outlets. Various media outlet posts are differentially weighted based on the visibility of the medium on which it was shared. For example, a mention of an article by a news outlet is worth 32 times the value of a tweet of an article. The complete weighting system can be viewed on the AAS website [11,13]. Concurrently, the National Institute of Health iCite function was queried to determine the number of citations accrued by each article in the analysis [14]. A Kruskal-Wallis test was used to assess the Altmetric score for COVID-19-related versus non-COVID-19-related articles. Articles in the COVID-19-related cohort were further characterized across multiple different variables, including the number of authors, country of origin, time from submission to publication, type of study design, quarter of the year it was published, and subspecialty of anesthesiology it pertained to. Statistical analysis was performed using the R statistical software version 4.0.2 (R Foundation for Statistical Computing, Vienna, Austria) with a predetermined level of significance set as a p-value of <0.05.

Results

**Figure 1:**
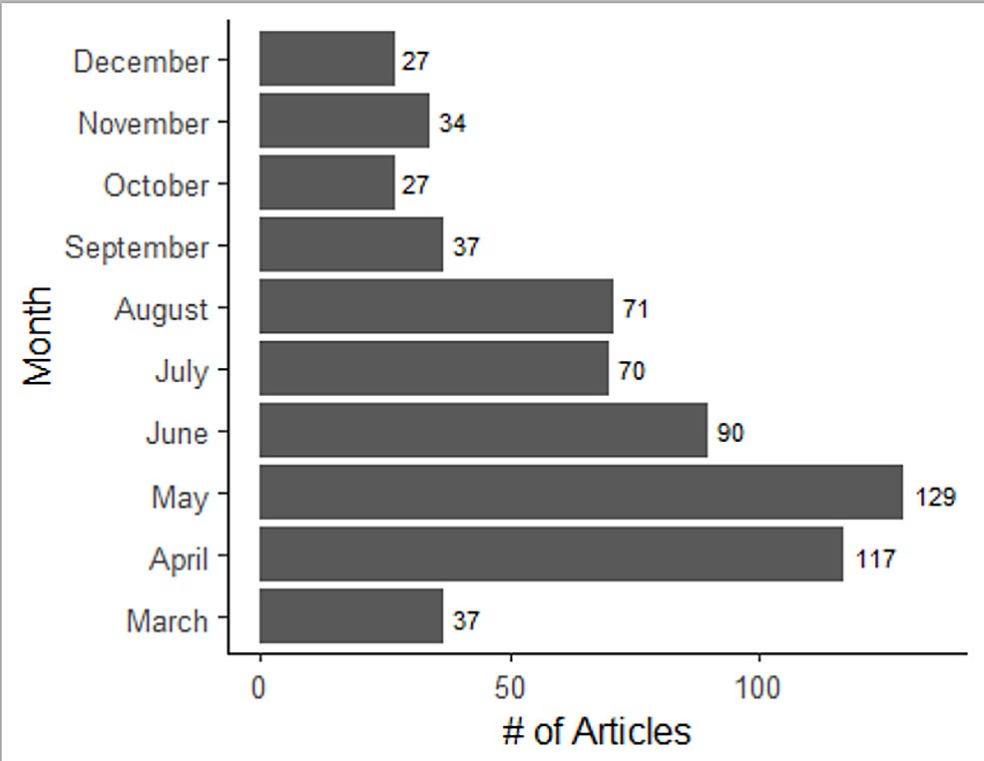
Total number of COVID-19-related articles published by month in 2020.

Of the 15 journals investigated, 676 (12%) of the 5,722 total articles published were related to the COVID-19 pandemic. Primary authors from the United States published the highest proportion (30%) of COVID-19-related articles, followed by authors from institutions in the United Kingdom (14%) and Canada (9%). The majority of articles were published in April (18%), May (20%), and June (14%) (Figure 1). Most articles were related either to general anesthesiology (48%) or critical care (39%) (Table 1). When examined based on the type of publication, most COVID-19-related articles were determined to be editorials (71%), followed by original research articles (21%). Of the original research articles, the majority were cross-sectional (55%) studies. The mean time from submission to publication for all articles with this data available (n = 465) was 41 days, with editorial articles having the quickest turnaround time (34 days for editorials versus 60 days for non-editorials, p < 0.001).

**Table 1:** Number of COVID-19-related articles published in each subspecialty of anesthesiology.

Specialty	Number of articles	Proportion (%)
General	312	48.3
Critical care	254	39.3
Regional/acute pain	29	4.5
Obstetrics	26	4
Cardiothoracic	11	1.7
Pediatric	9	1.4
Neuro	5	0.8

**Figure 2:**
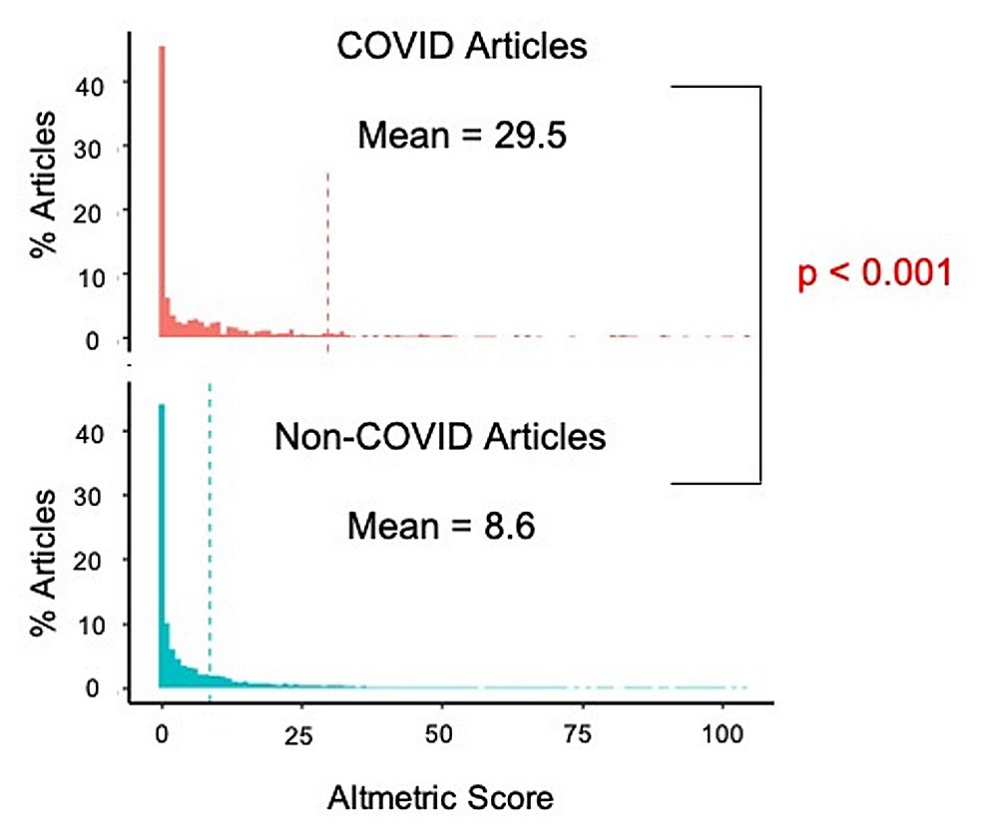
Mean Altmetric score for COVID-19-related articles versus non-COVID-19-related articles.

When compared with non-COVID-19-related articles, COVID-19-related articles had a significantly higher AAS (30 versus 9, p < 0.001) (Figure 2). Additionally, COVID-19-related articles published in quarter 1 (January to March) had a significantly higher AAS (79 versus 21 versus 12 versus 50, p < 0.001) when compared with COVID-19-related articles published during any other quarter of the year (Table 2). Of the COVID-19-related articles, original articles had the highest AAS when compared to editorials and guidelines (55 versus 21 versus 41, p < 0.002). There was no statistical difference in AAS across the types of original articles published, whether it was cross-sectional, review, survey, or case report (p = 0.83). The AAS for articles pertaining to general anesthesiology and critical care was higher than the other subspecialties; however, the association was not statistically significant (p = 0.65).

**Table 2:** Mean Altmetric score for COVID-19-related articles published in each quarter of 2020.

	Quarter 1 (N = 37)	Quarter 2 (N = 336)	Quarter 3 (N = 178)	Quarter 4 (N = 88)	Total (N = 639)	p-value
Altmetric score (mean ± SD)	79 (137)	21 (63)	12 (40)	50 (205)	26 (98)	<0.001

Regarding citations, COVID-19-related articles had more citations than non-COVID-19-related articles (7 versus 0.8, p < 0.001). Articles that were published earlier in the year had more citations (37.5 (quarter 1) versus 6.7 (quarter 2) versus 0.8 (quarter 3) versus 0.4 (quarter 4), p < 0.001). Overall, the articles had a median word count of 1,436 and referenced a mean of 17 sources.

Discussion

The hypothesis of this study was that the COVID-19-related literature in the anesthesiology community would gain more interest than non-COVID-19-related articles, and this hypothesis was supported by our data, specifically with regard to the AAS that was calculated for both types of articles. The COVID-19 pandemic sparked a great deal of interest in the medical community, specifically within the academic anesthesiology community. The AAS, a metric for the interest and attention that an article receives, was significantly higher for COVID-19-related articles. This highlights that COVID-19 research was of the utmost influence and interest to the anesthesia community during 2020. Furthermore, the AAS was the highest for articles discussing original research studies. The anesthesiology community was eager to learn about the effects of COVID-19 on their specialty, as made evident by the earliest published articles (January to March) receiving the highest levels of attention and dissemination (Table 2). This underscores the increased levels of discussion among academic anesthesiologists regarding the pandemic and the ramifications that it has on their specialty.

When compared to other specialties, anesthesiology had noticeably more literature published about COVID-19 than others [13,15,16]. In our study, COVID-19 literature represented 12% (676) of the total number of articles investigated, whereas a similar study conducted in plastic surgery literature identified that only 3.2% (220) of the total articles involved COVID-19 [13].

The median time to publication for the COVID-19 articles was 41 days. This quick turnaround is a stark contrast to other studies that showed that article publication time can vary from months to years [8]. A number of explanations exist, such as the need for new COVID-19 information as the pandemic progressed, as well as increased demand to publish COVID-19-related information in a timely manner. Of note, the greatest number of articles were published in April, May, and June 2020, which represented the early stages of the pandemic when knowledge was still relatively limited.

Our analysis revealed that COVID-19 articles accrued more citations compared with non-COVID-19 articles. This demonstrates that articles related to the COVID-19 pandemic not only were of heightened public interest but also were higher in impact [11,12]. Articles published earlier in the year accrued more citations than those published later in the year. This is most readily explained by the lag time between article publication and subsequent citation in the literature whereby articles published earlier have more time to accrue subsequent citations [9]. Given the life- and work-altering changes ushered in by the pandemic, it seems natural that these influences and impact would be reflected in the anesthesiology literature.

As observed in Table 2, the articles published in quarter 1 had the highest AAS, likely due to the increased interest in new information at the beginning of the pandemic. The next highest mean AAS (50) was in quarter 4, and this is when many of the original articles and guideline statements began to be published. Given that non-editorial articles had a mean time to publication of 60 days versus 34 days for editorials, it is expected that these articles were published later in the year. Knowing this information, it would be interesting for future studies to assess the impact of COVID-19 articles that are published with long-term follow-up of AAS and subsequent citations in the literature. Many of these articles will likely be original articles, which lend themselves to having a higher AAS; however, the interest in COVID-19 may not ever be as high as it was at the beginning of the pandemic. Further characterization of these trends is necessary to classify this relationship.

A large majority of the articles published were editorials, and these articles often served to share valuable experiences regarding the critical care management of COVID-19 patients and discussed the utilization of limited resources. These articles served as inspiration for other academic anesthesiologists to share their experiences in treating COVID-19 patients and very quickly prompted collaborations between institutions from many different countries across the globe, highlighting the unification of anesthesiologists internationally to address the COVID-19 pandemic.

When cases of COVID-19 began to rapidly rise in April 2020, the anesthesiology community responded, as over 51% of the COVID-19 articles were published in the second quarter of the calendar year (April to June) [3]. While there was no statistically significant difference in the AAS across subspecialties of anesthesiology, there was a large interest in the critical care aspect of anesthesiology, as 39% of all articles were related to critical care (Table 1). This is of particular importance as it highlights the unique value of anesthesiologists as both physicians in the operating room and the critical care setting. Decisive action and creativity have served as the cornerstones of anesthesiology during this pandemic, and in the face of a rapidly changing medical landscape, anesthesiologists have demonstrated ingenuity in their approach to patient care.

The largest limitation of this study is that there is not a strong control group to compare our results to. There are similar published data from other subspecialties that were affected by the COVID-19 pandemic (plastic surgery, orthopedic surgery, and oral-maxillofacial surgery); however, the most valuable data would be from subspecialties that are inherently more similar to anesthesiology (general surgery, internal medicine/critical care medicine, and pediatrics) [13,15,16]. Additionally, in this study, we elected to study the AAS as a continuous variable. Other analysis methods might reveal different trends that were outside the scope of this present study. Future studies can aim to highlight these comparisons as the data becomes available. Another limitation of this study is that we only analyzed articles published in English, which naturally excludes a fair number of articles from China, Italy, and other European countries that were affected by the pandemic.

## Conclusions

The response of the academic anesthesiology community to this worldwide pandemic was quick and decisive. As COVID-19 cases began to rapidly rise in both the United States and across the world, anesthesiologists responded swiftly with new high-impact research that would serve to guide our nation and world through one of its most challenging times. This response by the anesthesiology community underscores that while anesthesiologists are of value in the operating room, they also have tremendous value as critical care physicians.
